# Dietary carbohydrate quality index (CQI), cardio-metabolic risk factors and insulin resistance among adults with obesity

**DOI:** 10.1186/s12902-023-01420-4

**Published:** 2023-08-11

**Authors:** Negin Nikrad, Babak Hosseini, Azin Pakmehr, Ayda Zahiri Tousi, Abnoos Mokhtari Ardekani, Mahdieh Abbasalizad Farhangi, Reza Akhavan-Sigari

**Affiliations:** 1 Tabriz Health Services Management Research Center, Tabriz University of Medical Sciences, Tabriz, Iran; 2 Department of Surgery, School of Medicine, Laparoscopy Research Center, Shiraz University of Medical Sciences, Shiraz, Iran; 3 Tehran University of Medical Sciences, Tehran, Iran; 4 Razavi Cancer Research Center, Razavi Hospital, Imam Reza International University, Mashhad, Iran; 5 Endocrinology and Metabolism Research Center, Institute of Basic and Clinical Physiology Science, & Physiology Research Center, Kerman University of Medical Sciences, Kerman, Iran; 6 Department of Community Nutrition, Faculty of Nutrition, Tabriz University of Medical Sciences, Tabriz, Iran; 7 Department of Neurosurgery, University Medical Center Tuebingen, Tuebingen, Germany; 8 Department of Health Care Management and Clinical Research, Collegium Humanum Warsaw Management University, Warsaw, Poland

**Keywords:** Carbohydrate quality index, Obesity, Metabolic disorders, Insulin resistance

## Abstract

**Background:**

Metabolic syndrome (MetS), as a cluster of cardiometabolic risk factors, is a global public health concern due to its increasing prevalence. Considering the previous evidence of the association between carbohydrate quality and cardiometabolic risk factors, our study was aimed to evaluate any possible association between carbohydrate quality index (CQI) and cardiometabolic risk factors among obese adults.

**Methods:**

In this cross-sectional study, 336 apparently healthy individuals with obesity were participated. Dietary intake was assessed by a semi-quantitative Food Frequency Questionnaire (FFQ), including 168 food items validated for the Iranian population. CQI was calculated with three components of solid carbohydrates to total carbohydrates ratio, dietary fiber intake, and dietary glycemic index (GI). Body composition was determined by bioelectrical impedance analysis (BIA). Blood pressure was measured by sphygmomanometer and enzymatic methods were used to evaluate serum lipid, glucose, and insulin concentrations.

**Results:**

Subjects in the third quartile of CQI had significantly lower systolic blood pressure (SBP) (P = 0.03) and diastolic blood pressure (DBP) (P = 0.01). Participants in the higher quartiles of CQI had more intake of energy, carbohydrates, fat, saturated fatty acid (SFA), and mono-saturated fatty acid (MUFA) (P < 0.05). Moreover, the homeostasis model assessment of insulin resistance (HOMA-IR) was decreased in the second quartile of CQI [odds ratio (OR) = 0.146, P = 0.01) after adjustment for age, body mass index (BMI), sex, physical activity, socioeconomic status (SES) and energy intake.

**Conclusion:**

According to our findings, a higher quality of dietary carbohydrates, determined by CQI, could be associated with a lower risk of hypertension.

## Introduction

Metabolic syndrome (MetS), as a cluster of cardiometabolic risk factors, is defined predominantly by central obesity, insulin resistance (IR), dyslipidemia, hypertension, and hyperglycemia [1–3]. It consists of atherogenic dyslipidemia, high blood pressure, IR, high blood glucose, and a pro-thrombotic and pro-inflammatory state [4]. MetS prevalence has reached 20–25% in the adult population of developed countries, and its incidence is still increasing over time [5, 6]. MetS now affects 30.4% of the Iranian population, with a considerable rising trend [7]. Recent research has revealed a relationship between modifying lifestyle factors, particularly dietary habits and prevention of MetS [8]. Since the fundamental goal of MetS treatment is to minimize the risk of cardiovascular diseases (CVDs), previous dietary guidelines, such as those given by the national cholesterol education panel (NCEP)-adult treatment panel (ATP) III, and the American heart association (AHA) have mostly focused on modifying the macronutrient content of the diet [9]. Although dietary carbohydrates are the only macronutrients that directly affect blood sugar and insulin levels, the quality of carbohydrates appears to play a more critical role in the prevention of chronic disease rather than total carbohydrate as a percentage of dietary energy [10]. Associations between source of dietary carbohydrate such as dietary fiber [11–13], glycemic index (GI) or glycemic role (GL) [14–16] and the MetS incidence have been previously reported. Because dietary carbohydrates are a heterogeneous class of dietary nutrients, it is necessary to establish a new index for measuring the quality of dietary carbohydrates; carbohydrate quality index (CQI) was developed to provide a more comprehensive perspective of dietary carbohydrate quality by taking dietary total fiber consumption, dietary GI, whole grains-to-total grains ratio, and the ratio of solid carbohydrates to total carbohydrates into account [17].

The association between CQI and several cardio-metabolic risk factors has been investigated before. A better quality of dietary carbohydrates measured by the CQI was in a significant inverse association with the incidence of CVD [18].

An inverse association between dietary CQI and general and abdominal obesity [17, 19] and odds of MetS [20] was also recognized. Analyses of more than 120 000 adults from 16 cohort studies suggested that obesity has been associated with a twofold increase in the risk of developing cardiometabolic multi-morbidity [21, 22]. Obesity increases the risk of dyslipidemia and systemic inflammation, which has been linked to the onset of diabetes and vascular disease [23]. Meanwhile, in recent years, the frequency and prevalence of general and visceral obesity have increased in Iranian people [24, 25]. Given the high consumption of carbohydrates among the Iranian population, the mean percentage of total energy received from carbohydrates is 65% and the amount of total carbohydrates consumed from bread and white rice are 34.2 and 14.8%, respectively [10], it is necessary to evaluate the association between quality of carbohydrates and components of MetS and cardiometabolic risk factors among apparently healthy individuals with obesity in Iranian adults. Therefore, we examined the association between quartiles of CQI and cardio-metabolic risk factors among Iranian population.

## Methods and materials

### Participants

A random sample of individuals with obesity [body mass index (BMI) > 30 kg/m^2^] were recruited from the previous projects [26, 27]. 336 individuals aged between 20 and 50 years old were participated through public announcements. In the current study the exclusion criteria were as follows: pregnant, lactating, or menopausal women, gastric bypass and other weight-loss surgeries, history of cardiovascular disease, cancer, hepatic or renal disorders, diabetes mellitus, and using any drugs or supplements affecting weight. All subjects read and signed an informed consent form and the Ethics Committee of Tabriz University of Medical Sciences, Iran (Identifier: IR.TBZMED.REC.1398.460 and IR.TBZMED.REC.1396.768).

### General characteristics

The socio-demographic questionnaire obtained information about sex, age, education attainment, smoking status, marital status, medical histories, occupation, and family size. Then with the extracted information, socioeconomic status (SES) score was calculated using the following items: educational status, occupational position, house ownership, and family size [28, 29]. We assumed education as a categorical variable where individuals should report their highest level of education. This variable was recorded on a 5-point scale ranging from 0 to 5 (illiterate: 0, less than diploma: 1, diploma and associate degree: 2, bachelors: 3, masters: 4 and higher: 5). Occupational status for females was categorized into five groups (housewife, employee, student, self-employed, and others), and also occupational status of male participants was categorized as follow: unemployed: 1, worker, farmer and rancher: 2, others: 3, employee: 4 and self-employed: 5. Accordingly, participants were categorized as ≤ 3, 4–5, ≥ 6 in terms of family size. Besides, they were given scores of 1 if they were tenants and 2 if they were the landlord. Then, the participants were classified into 3 categories of low, medium and high, based on the total SES score, which has a score between 1 and 15. The appetite status of the participants was evaluated using a visual analog scale (VAS) [30]. The VAS was calculated by marking a 100-mm line at each end of the line with the opposite words “I’m not at all hungry” and “I have not been so hungry”. Also, using the same questionnaire, it was asked about the cravings for sweet, salty, and fatty foods, satiety, fullness, and future food intake. A short version of the International Physical Activity Questionnaire (IPAQ) was used to evaluate the physical activity level of participants [31, 32]. Blood pressure was measured with a standard mercury sphygmomanometer twice after at least 15 min of rest in one arm, and then the average of the two measurements was used for analysis.

### Anthropometric assessments

Height and weight were determined to the nearest 0.5 cm and 0.1 kg using a wall-mounted stadiometer and a Seca scale (Seca co., Hamburg, Germany), respectively. Bioelectrical impedance analysis (BIA) was used to determine the body composition (Tanita, BC-418 MA, Tokyo, Japan) as a quick, non-invasive, and reliable way to measure body composition [33]. This device represents body fat percentage, fat mass (FM), fat free mass (FFM), and predicted muscle mass. Measurement of body composition by BIA and weight on a scale was done without shoes and with minimal clothing. Waist circumference (WC) was measured using a tape measure to the nearest 0.1 cm at the midpoint between the lower costal margin and the iliac crest, and hip circumference (HC) was measured the part that yields the maximum diameter over the buttocks and was recorded to the nearest 0.1 cm.

### Dietary assessment

Dietary intake of participants was collected using a validated 168-item semi-quantitative food frequency questionnaire (FFQ), for Iranian population [34]. A trained nutritionist collected the information on the frequency and amount of consumption of each food item on a daily, weekly, and monthly basis through a face-to-face interview. Then, the reported frequency and portion sizes for each 168 food item were converted to gram using household measures.

### Calculation of carbohydrate quality index (CQI)

The CQI was calculated by adding together the following three components, solid carbohydrates to total carbohydrates ratio (including solid and liquid carbohydrates), dietary fiber intake (g/day) and dietary GI [35]. The initial score includes whole grains, but due to very low consumption of cereal and whole grains in the eating habits of Iranians [36, 37] and considering that the ratio of whole grains to total grains tends to zero, we decided not to include whole grains in the CQI score. Glycemic index of each food item was obtained the glycemic index of Iranian food book. Liquid carbohydrates were calculated by summing the total amount of dietary carbohydrates from all sugar-sweetened drinks and fruit juices, any dairy product including milk, yogurt and milkshakes or plant-based milk products, as well as tea, coffee and alcoholic drinks [38], while the carbohydrate content of the remaining food items were considered solid carbohydrates [39]. For each of mentioned three components, participants were classified into quintiles and were given a value (score one to five) according to each quintile and descending scoring was used for GI quintiles. Then, the overall CQI was calculated from the sum of the scores for the three components (from 3 to 15).

### Biochemical assessment

10 ml of venous fasting blood from each participant was obtained for the biochemical analysis, centrifugation was used to separate the serum and plasma samples for 10 min at 4 °C at 4,500 rpm. Aliquots were frozen at 70 °C until analysis. Total serum cholesterol (TC), triglycerides (TG), high-density lipoprotein cholesterol (HDL-C), and fasting blood glucose (FBG) were measured using commercial kits according to the manufacturer’s instructions (Pars Azmoon, Tehran, Iran). Blood insulin levels were also assessed by a commercial kit (Bioassay Technology Laboratory, Shanghai Korean Biotech, Shanghai City, China). The Friedewald equation [40] was used to calculate the amount of low-density lipoprotein cholesterol (LDL-C). The Homeostasis Model Assessment of Insulin Resistance (HOMA-IR) was calculated by fasting insulin (IU/ml)/22.5 fasting glucose (mmol/l), and the Quantitative Insulin Sensitivity Check Index (QUICKI) was determined by 1/ [log fasting insulin (U/mL) + log glucose (mmol/L) [41].

### Statistical analysis

The data were analyzed using Statistical Package for Social Sciences (SPSS, version 21.0; SPSS Inc, Chicago IL). Using histogram charts and the Kolmogorov-Smirnov test, the variables’ normality was examined. For quantitative data that were normally distributed, the distribution was expressed as mean (SD), and for qualitative data as frequency (%). The differences in discrete and continuous variables across different quartiles of CQI were compared using chi-square test and one-way analysis of variance (ANOVA), respectively. The association between the CQI quartiles and biochemical variables was analyzed using multinomial logistic regression to estimate odds ratios (ORs) and 95% confidence intervals (CIs) for the risk of elevated cardiometabolic risk components including blood pressure, glycemic profile and blood lipid profiles across CQI quartiles in three multivariable-adjusted models. Linear trends across quartiles of CQI were assessed by modeling the value of median in each quartile as a continuous variables in the regression models.

## Results

In the present study, 336 individuals with obesity (with a mean BMI of 32.62 ± 4.80 kg/m2) aged 40.78 ± 9.23 years old were participated. Table 1 presents an overview of the general demographic characteristics of the study participants according to CQI quartiles. It is apparent from this Table that while socio-economic status and gender were significantly different between CQI quartiles (p > 0.05, P for trend, 0.01 and < 0.01 respectively), also an increasing trend of age was observed in higher quartiles of CQI (P for trend, 0.03). Results reported no significant difference in age, BMI, WC, FM, FFM, WHR, appetite, and basal metabolic rate (BMR) among different quartiles of CQI. However, regarding the comparison of biochemical parameters of study participants in different quartiles of CQI, as shown in Table 1, there were no statistical differences amongst CQI quartiles except for SBP (P < 0.05) and DBP (P < 0.02). This significant difference in CQI quartiles specifically was related to the reduction of SBP and DBP levels in the third quartile of CQI compared to the first quartile (P = 0.03 and 0.01 respectively). However, the decreasing trend in SBP and DBP levels was not statistically significant. As Tables 2 and 3 present, there is a significant difference in dietary intakes of CQI components (P < 0.001), meat, fish poultry (MFP) intake, dairy, and grains (P < 0.05) across different quartiles of CQI. In addition, Table 3 compares dietary intakes of energy and macronutrients, and shows remarkably increased intake of energy, carbohydrate, fat, SFA, and MUFA in higher quartiles of CQI (P < 0.05) after adjustment for potential confounders. ORs and 95% CIs for cardiometabolic risk factors by quartiles of CQI are presented in Table 4. As shown, after adjusting for age and sex, individuals in the third quartile of CQI had lower DBP (OR = 0.949, P = 0.03). HOMA-IR showed a significant reduction in the second quartile of CQI in model III (OR = 0.146, P = 0.01). No significant relationship was observed between CQI and SBP, FBS, LDL, HDL, TG, and QUICKI in the crude and multivariable adjusted models.

**Table 1:** General demographic characteristics of study participants by quartiles of CQI

Variable	Quartiles of CQI								P* value	P trend
	1st ^(N=86)^		2nd ^(N=85)^		3rd ^(N=84)^		4th ^(N=81)^			
	Mean	SD	Mean	SD	Mean	SD	Mean	SD		
Age (y)	39.54	9.24	40.18	9.00	41.71	9.48	41.13	8.82	0.40	**0.03**
P	-		0.96		0.37		0.71			
Gender (% Male)	64.64	48.05	66.30	47.52	44.70	50.01	50	50.42	< 0.01**	**< 0.01**
P	-		0.99		**0.03**		0.26			
BMI (kg/m^2^)	31.88	5.25	32.44	4.44	33.01	4.67	33.72	4.61	0.10	0.96
P			0.85		0.38		0.08			
WC (cm)	106.51	9.05	107.35	10.02	105.54	9.72	107.55	9.70	0.54	0.52
P	-		0.93		0.90		0.91			
FM (%)	33.00	11.45	34.19	7.68	33.27	7.89	34.53	9.69	0.83	0.08
P	-		0.92		0.99		0.87			
FFM (%)	65.30	12.22	62.34	11.64	60.55	13.03	61.19	12.49	0.27	0.08
P	-		0.65		0.25		0.43			
WHR	0.94	0.076	0.93	0.084	0.92	0.072	0.93	0.06	0.43	0.88
P	-		0.91		0.37		0.76			
SES	10.76	2.48	10.30	2.26	9.64	2.27	9.17	2.79	**0.01**	**0.01**
P	-		0.78		0.12		**0.01**			
Appetite	33.90	8.72	32.15	9.27	35.21	9.01	33.05	8.69	0.35	0.29
P	-		0.77		0.89		0.97			
BMR (Kcal)	8285.23	1429.68	7813.42	1720.35	7715.11	1533.84	7785.30	1479.51	0.29	0.88
P	-		0.45		0.29		0.46			
SBP (mmHg)	125.63	13.16	122.38	14.57	119.11	20.83	122.96	14.77	0.05***	0.53
P	-		0.50		**0.03**		0.74			
DBP (mmHg)	83.60	9.92	81.75	10.77	78.51	14.43	81.76	9.87	0.02***	0.86
P	-		0.67		**0.01**		0.76			
FBS (mg/dl)	95.12	26.47	90.84	12.26	92.37	18.88	92.05	14.64	0.48***	0.96
P	-		0.42		0.77		0.76			
TC (mg/dl)	193.31	39.54	192.70	35.84	190.62	37.55	189.40	33.22	0.90***	0.06
P	-		0.99		0.96		0.91			
TG (mg/dl)	167.60	84.65	149.78	121.01	141.09	77.90	138.10	76.57	0.15***	0.22
P	-		0.55		0.22		0.21			
HDL (mg/dl)	43.33	9.42	42.90	10.06	44.44	8.88	43.66	9.95	0.74***	0.60
P	-		0.99		0.86		0.99			
LDL (mg/dl)	124.53	34.30	124.83	31.16	121.62	32.04	122.54	30.23	0.89***	0.55
P	-		0.99		0.92		0.98			
Insulin (mIU/l)	17.52	12.68	15.22	9.93	17.48	19.73	13.17	7.57	0.27***	0.95
P	-		0.76		0.99		0.32			
HOMA-IR	4.20	3.38	3.44	2.55	4.09	4.34	3.02	1.80	0.17***	0.71
P	-		0.52		0.99		0.29			
QUICKI	0.32	0.037	0.33	0.033	0.32	0.036	0.33	0.037	0.36***	**0.01**
P	-		0.75		0.86		0.28			

CQI, carbohydrate quality index; BMI, Body mass index; WC, Waist Circumference; FM, Fat Mass; FFM, Fat Free Mass; WHR, waist-to-hip ratio; BMR, Basal Metabolic Rate; SES, socio-economic status; SBP, Systolic Blood Pressure; DBP, Diastolic Blood Pressure; FBS, fasting blood glucose; TC, Total Cholesterol; TG, Triglyceride; HDL-C, High Density Lipoprotein Cholesterol; LDL-C, Low Density Lipoprotein Cholesterol; HOMA-IR, Homeostatic Model Assessment for Insulin Resistance; QUICKI, Quantitative Insulin sensitivity Check Index; all data are mean (± SD) except gender, that is presented as percent of males in each group. P* values derived from One-Way ANOVA with Tukey’s post-hoc comparisons. P** values derived from chi-squared test. P*** values derived from One-Way ANOVA with Tukey’s post-hoc comparisons after adjustment for confounders (age, gender, BMI, PA and kcal). P**** for differences among CQI quartiles from the first quartile (ANOVA with Tukey’s post hoc test)

**Table 2:** Dietary intakes of CQI components according to quartiles of CQI

Variable	Quartiles of CQI								P* value
	1st ^(N=86)^		2nd ^(N=85)^		3rd ^(N=84)^		4th ^(N=81)^		
	Mean	SD	Mean	SD	Mean	SD	Mean	SD	
Liquid carbohydrate	126.82	124.30	75.94	117.49	71.69	104.02	27.09	26.23	**< 0.001**
Solid carbohydrate	251.60	153.22	349.25	158.53	407.10	143.85	549.06	195.02	**< 0.001**
Glycemic index (GI)	62.71	30.73	50.74	14.03	44.55	14.61	39.58	8.174	**< 0.001**
Fiber (g/d)	43.31	16.24	55.12	23.092	75.57	38.10	106.43	47.90	**< 0.001**

CQI, carbohydrate quality index. * P-values are derived from one-way ANOVA test

**Table 3:** Food groups intake of study participants by quartiles of CQI

Variable	Quartiles of CQI									
	1st ^(N=86)^		2nd ^(N=85)^		3rd ^(N=84)^		4th ^(N=81)^		P* value	P** value
	Mean	SD	Mean	SD	Mean	SD	Mean	SD		
Fruits (g/d)	3.31	1.77	3.69	2.31	4.47	2.79	5.37	4.82	**0.01**	0.93
Vegetables (g/d)	3.63	1.75	3.86	2.25	3.77	2.41	4.16	2.61	0.75	0.07
MFP (g/d)	3.14	1.49	3.16	1.51	3.82	2.08	3.76	1.75	**0.03**	**0.001**
Dairy (g/d)	2.36	1.55	2.16	1.18	2.11	1.36	1.64	1.01	0.08	**< 0.001**
Grains (g/d)	11.28	5.09	11.72	4.76	14.84	6.56	19.88	8.04	**< 0.001**	**< 0.001**
Energy (kcal/d)	2580.53	826.37	2897.07	1081.69	3173.69	1027.79	3705.29	1242.42	**< 0.001**	**< 0.001**
CHO (%)	56.27	5.94	57.31	6.64	57.65	7.21	61.21	6.98	**0.007**	**0.002**
Protein (%)	13.31	1.63	13.07	1.88	13.27	2.10	12.34	2.13	0.08	0.26
Fat (%)	33.13	5.84	32.37	7.17	31.54	7.46	29.05	6.36	**0.04**	**0.002**
Cholesterol (mg/d)	282.51	144.30	281.59	155.48	322.23	284.38	310.22	194.52	0.44	0.14
SFA (g/d)	27.65	12.37	28.61	14.76	29.68	12.95	32.89	20.68	0.18	**< 0.001**
MUFA (g/d)	29.06	13.22	33.07	16.13	34.01	15.47	39.11	21.40	**0.003**	**0.04**
PUFA (g/d)	18.01	9.22	22.33	12.91	23.86	13.05	28.30	16.07	**< 0.001**	0.92

MFP, meat, fish and poultry; CHO, carbohydrate, SFA, saturated fatty acids, MUFA, mono-unsaturated fatty acids; PUFA, polyunsaturated fatty acids. All data are mean (± SD). P* values derived from unadjusted ANCOVA P** values derived from ANCOVA after adjustment for confounders (age, gender, BMI, PA and energy intake)

**Table 4:** Odd’s ratio (OR) and confdence interval (CI) for biochemical variables of study participants by quartiles of CQI

Variable		Quartiles of CQI							
		1st ^(N=86)^	2nd ^(N=85)^		3rd ^(N=84)^		4th ^(N=81)^		p-trend
			OR(CI)	P-value	OR(CI)	P-value	OR(CI)	P-value	
SBP (mmHg)	Model I	**1** **REF**	0.989 (0.956–1.024)	0.53	0.996 (0.961–1.032)	0.82	0.989 (0.953–1.027)	0.56	0.32
	Model II		0.986 (0.952–1.022)	0.44	0.995 (0.959–1.032)	0.79	0.989(0.952–1.027)	0.56	0.49
	Model III		0.997 (0.948–1.049)	0.91	0.998 (0.948–1.051)	0.95	0.995 (0.940–1.053)	0.85	0.77
DBP (mmHg)	Model I	**1** **REF**	1.000 (0.954–1.048)	0.99	0.956( 0.911–1.004)	0.07	1.016 (0.964–1.070)	0.55	0.86
	Model II		0.995 (0.948–1.045)	0.85	0. 949( 0.902-0.997)	**0.03**	1.004 (0.952–1.059)	0.87	0.42
	Model III		0.995 (0.936–1.058)	0.87	0.946 (0.887–1.008)	0.08	1.024 (0.956–1.096)	0.50	0.73
FBS (mg/dl)	Model I	**1** **REF**	1.004 (0.968–1.040)	0.84	1.002 (0.969–1.035)	0.92	1.011 (0.969–1.055)	0.62	0.27
	Model II		1.001 (0.964–1.039)	0.96	1.004 (0.970–1.039)	0.82	1.009 (0.964–1.055)	0.71	0.58
	Model III		1.039 (0.978–1.104)	0.21	0.997 (0.943–1.054)	0.91	1.001 (0.940–1066)	0.96	0.94
TC (mg/dl)	Model I	**1** **REF**	1.019 (0.982–1.057)	0.32	1.002 (0.977–1.028)	0.85	1.000 (0.976–1.025)	0.97	0.61
	Model II		1.021 (0.983–1.060)	0.29	1.004 (0.979–1.030)	0.75	1.002 (0.977–1.027)	0.89	0.65
	Model III		1.020 (0.991–1.037)	0.12	1.021 (0.992–1.038)	0.10	1.019 (0.995–1.038)	0.09	0.23
TG (mg/dl)	Model I	**1** **REF**	0.995 (0.987–1.003)	0.18	0.998(0.992–1.004)	0.52	0.995 (0.989–1.002)	0.15	0.65
	Model II		0.994 (0.987–1.002)	0.16	0.998 (0.991–1.004)	0.47	0.995 (0.988–1.001)	0.12	0.75
	Model III		0.995 (0.987–1.004)	0.31	0.992 (0.983–1.002)	0.11	0.996 (0.987–1.006)	0.47	0.39
HDL (mg/dl)	Model I	**1** **REF**	0.998 (0.948–1.052)	0.94	1.018 (0.974–1.065)	0.42	1.002 (0.956–1.050)	0.94	0.31
	Model II		0.998 (0.944–1.054)	0.92	1.009 (0.962–1.058)	0.47	0.990 (0.941–1.041)	0.68	0.37
	Model III		0.982 (0.925–1.042)	0.53	0.974 (0.917–1.035)	0.39	0.954 (0.892–1.021)	0.17	0.47
LDL (mg/dl)	Model I	**1** **REF**	0.990 (0.954–1.028)	0.60	1.002 (0.976–1.030)	0.85	1.005 (0.978–1.032)	0.71	0.41
	Model II		0.998 (0.951–1.027)	0.53	1.000 (0.973–1.028)	1.00	1.003 (0.976–1.031)	0.83	0.51
	Model III		0.993 (0.952–1.021)	0.58	1.001 (0.975–1.031)	0.93	1.002 (0.975–1.031)	0.86	0.63
Insulin (mIU/l)	Model I	**1** **REF**	1.067 (0.897–1.269)	0.46	1.019 (0.887–1.171)	0.79	1.029 (0.817–1.296)	0.80	0.23
	Model II		1.063 (0.890–1.270)	0.50	1.031 (0.893–1.191)	0.67	1.017 (0.785–1.317)	0.89	0.67
	Model III		1.495 (0.095–1.804)	0.07	0.971 (0.716–1.318)	0.85	0.999 (0.674–1.483)	0.99	0.91
HOMA-IR	Model I	**1** **REF**	0.694 (0.324–1.486)	0.34	0.986 (0.536–1.815)	0.96	0.724 (0.290–1.810)	0.49	0.32
	Model II		0.690 (0.315–1.513)	0.35	0.919(0.489–1.728)	0.79	0.699 (0.25–1.927)	0.48	0.56
	Model III		0.146 (0.032–0.661)	**0.01**	1.019 (0.367–2.825)	0.97	0.418 (0.09–1.824)	0.24	0.70
QUICKI	Model I	**1** **REF**	0.366 (1.557E-8-8616912.895)	0.90	3873.747 (0.002-6453005815)	0.25	0.407 (6.192E-10-267844979.0)	0.93	0.11
	Model II		0.150 (4.525E-9-4948759.073)	0.83	3607.057 (0.001-9175392150)	0.27	0.014 (2.752E-12-71361142.09)	0.70	0.14
	Model III		0.000 (1.874E-22-2.520E + 14)	0.69	0.038 (5.737E-18-2.537E + 14)	0.86	5.879E-21 (2.663E-43-129.757)	0.07	0.74

SBP, Systolic Blood Pressure; DBP, Diastolic Blood Pressure; FBS, fasting blood glucose; TC, Total Cholesterol; TG, Triglyceride; HDL-C, High-Density Lipoprotein Cholesterol; LDL-C, Low-Density Lipoprotein Cholesterol; HOMA-IR, Homeostatic Model Assessment for Insulin Resistance; QUICKI, Quantitative Insulin sensitivity Check Index; OR, odds ratio; CI, confidence interval. The multivariate multinomial logistic regression was used for the estimation of ORs and confidence interval (CI). Model I: crude, Model II: adjusted for age and sex, and Model III: adjusted for age, BMI, sex, physical activity, SES, and energy intake

## Discussion

The present cross-sectional study, as far as we know, is the first study to evaluate the possible link between carbohydrate quality index (CQI) and cardio-metabolic components. Our findings suggest that the levels of systolic and diastolic blood pressure reduced significantly in the third quartile of CQI. Although the trend of this reduction was not statistically significant, but, it can be considered that a higher index of carbohydrate quality might have a significant clinical impact in reducing blood pressure levels. The blood pressure- lowering effects of higher CQI quartiles can be attributed to increased fiber consumption and decreased GI in higher CQI quartiles. This finding was in agreement of the positive correlation between the CQI and fiber, and the negative correlation of CQI with the dietary GI previously reported in a case-control study from Ghana among 124 patients with T2DM [42]. A meta-analysis of 37 prospective observational studies showed that diets with a high GI or GL independently increased the risk of T2DM, CVD, gallbladder disease, breast cancer, and all diseases combined [43–45]. Another meta-analysis study that included 14 trials, consisting of 1097 healthy individuals, revealed that a lower glycemic potential of a diet may lead to significant reduction in blood pressure [46].

**Fig. 1:**
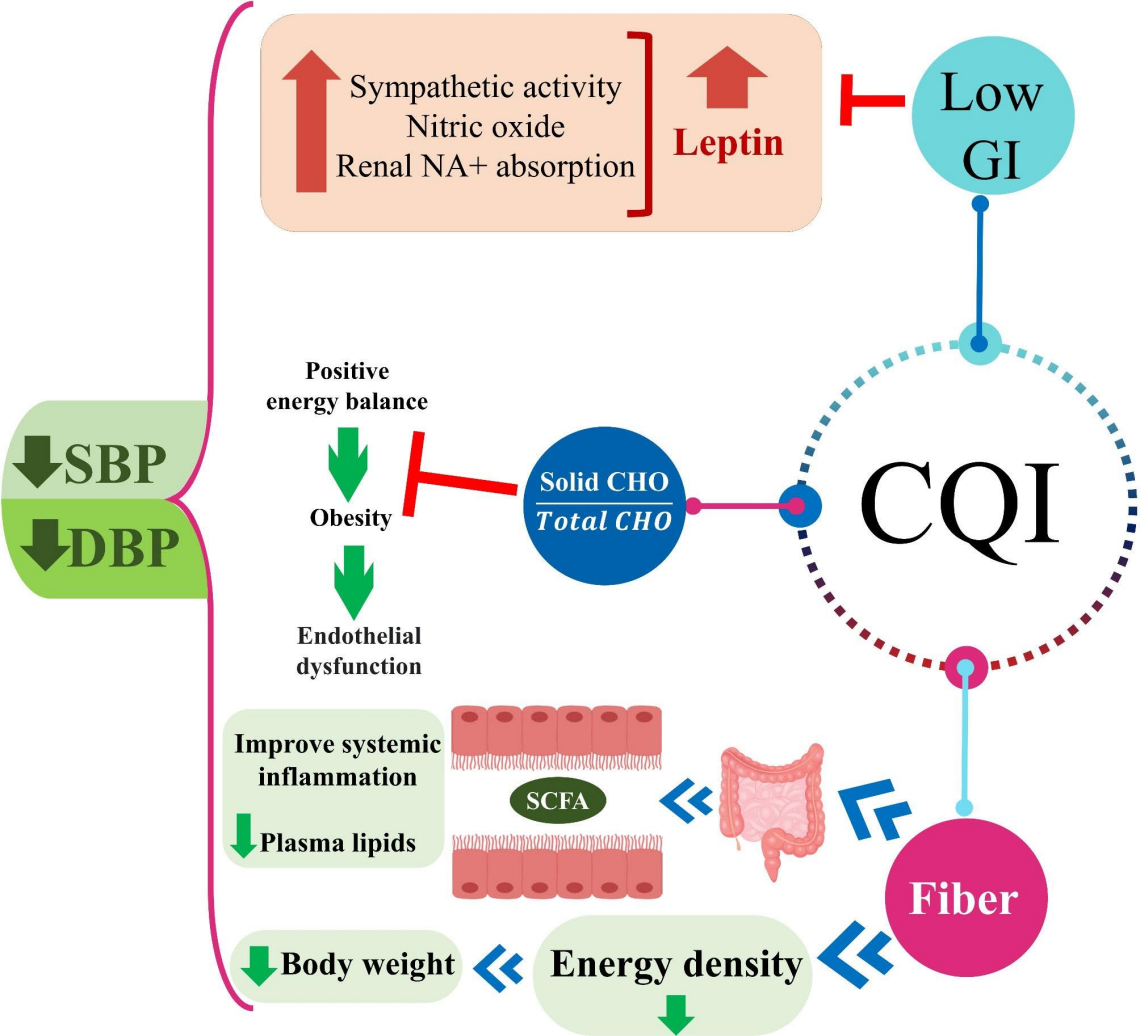
Mechanistic pathways of the possible effects of carbohydrate quality index (CQI) on blood pressure. Abbreviations; CQI, carbohydrate quality index; GI, glycemic index; CHO, carbohydrate; SCFA, short chain fatty acids, SBP, systolic blood pressure; DBP, diastolic blood pressure

A multitude of mechanisms may link dietary fiber to hypertension; one possible mechanism is probably related to the fact that some fibers contain polysaccharides, which are digested by gut bacteria to produce short-chain fatty acids (SCFA) [47], SCFAs have a significant effect on blood pressure regulation, since hypertension is often accompanied by a reduction in SCFA production [48, 49]. Dietary fiber-induced weight reduction has also been proposed as a potential mechanism of blood pressure regulation [50, 51]. On the other hand the adverse relationship between increasing dietary GI and changes in plasma leptin suggests that GI may play a role in controlling blood pressure [52, 53]; t has been demonstrated that chronic hyperleptinemia raises blood pressure [54, 55], that could be associated with the stimulation of the sympathetic nervous system, along with the impairment of natriuresis and nitric oxide (NO) suppression [56]. In addition, a cross-over study revealed that while a comparable solid carbohydrate causes appropriate dietary adjustment, increasing the intake of a liquid carbohydrate encourages positive energy balance [57] (Fig. 1). Consistent with our findings, Clar C et al. [58]. indicated that there was no convincing evidence of an effect of a low-GI diet on blood pressure, serum lipids, or cardiovascular events. In another study obtained from the Health Survey of São Paulo, no association was found between GI, GL, and MetS [59]. Dong Y et al. showed that total and soluble fiber consumption were found to be inversely related to SBP and DBP and also lower consumption of all forms of dietary fiber was significantly linked to higher insulin levels and HOMA-IR, regardless of calorie intake, BMI, or physical activity [60]. The findings of this research in terms of HOMA-IR levels were in agreement with our findings, we showed that HOMA-IR levels were lower in the second CQI quartile and also we observed a statistically non-significant but clinically significant decreasing trend of HOMA-IR was observed across quartiles of CQI. Da Rocha CM et al. [61] evaluated the association of dietary indicators of carbohydrate quality with markers of glycemic control and demonstrated that carbohydrate quality values were linked to biomarkers of glycemic homeostasis; they suggested that dietary GI index was better than GL in predicting insulinemia and consequently HOMA-IR, independent of weight status. Refined grains, such as bread and white rice, are two main sources of total calories in the Iranian diet, accounting for 55–60% of total calories intake [62], which generally have a high GI [63] and may be a risk factor for the development of MetS among Iranian population [64]. Furthermore, Iranians consume around 40% less cereal and whole bread than other countries [36, 37].

Bulló M et al. [42]. found that GI, GL, and CQI were associated with specific metabolomic profiles including choline, cotinine, γ –butyrobetaine, kynurenic acid and etc. that were related to a potential favorable cardio-metabolic risk in 1833 participants with overweight/obesity. Our findings indicated no significant relationship between BMI and WC with CQI. Consistent with our findings Suara S et al. examined the relationship between dietary CQI and odds of general and abdominal obesity in women within the ages of 18–59 years and the results showed that consumption of diets with high CQI value could be associated with reducing the risk of general and abdominal obesity [17, 65].

The evidence from this study suggests no significant relationship between CQI and FBS, TC, TG, HDL-C, LDL-C, and QUICKI. This finding supports previous findings of the Majdi M et al. study [66] that demonstrated a non-significant association between CQI and MetS and its components before and after adjustment for potential confounders. However, in a case-control study among T2DM patients, after an adjustment for potential cofounders, the CQI was negatively associated with WC, SBP, DBP and TG, and positively associated with HDL-C was shown [42].

Appetite regulation can be attributed to dietary macronutrients composition [67, 68]. A large-scale, long-term study concluded that over a period of three years, higher total carbohydrate, GL, and total fiber levels (but not GI) had been associated to increased participants hunger or appetite [69]. Additionally, studies have found that dietary fiber intake is linked to greater satiety and reduced energy consumption [70, 71]. Therefore, it can be concluded that the quality and quantity of dietary carbohydrate intake can be related to appetite [69]. However, in this study, no significant difference was observed in the appetite of participants in different quartiles of CQI.

Several number of limitations in the current study should be addressed here; firstly, due to the study’s cross-sectional design, any causal inference is challenging. Longitudinal researches are advised in the future to better define the cause and effect association; secondly, another possible cause of bias is that the FFQ used in this research was not designed particularly to assess CQI although it was a valid and reliable FFQ for dietary assessment of Iranian population, third limitation is the possibility of a memory-based (recall) bias of study participants in the use of questionnaires and fourth, since our study was conducted in Tabriz and Tehran cities of Iran, due to geographical variation, generalizing the results of the current study to other regions of the country should be done with caution. However, the relatively large sample size of the current study that examines the association of cardiometabolic risk variables and MetS components among obese individuals considering multiple adjustment for potential confounders in three models, are strengths of the current study.

## Conclusion

The results of this study indicate that individuals in the third quartile of CQI have lower systolic and diastolic blood pressure levels; therefore, it is possible to assume that a higher CQI might have a substantial therapeutic impact in lowering blood pressure levels, even though the trend was not significantly meaningful. Also, participant in higher quartiles of CQI have higher intake of solid carbohydrate, fiber and low-GI foods. No significant relationship between FBS, TC, TG, HDL-C, LDL-C, and QUICKI with CQI were observed in present cross-sectional study. Further longitudinal analysis are warranted to better elucidate the causality and underlying mechanisms.

## Data Availability

The datasets generated and/or analyzed during the current study are not publicly available due to privacy and ethical considerations, but can be available from the corresponding author on reasonable request.
